# A double-blind, placebo-controlled trial of Fennel *(Foeniculum vulgare)* on menopausal symptoms: A high placebo response

**DOI:** 10.4274/jtgga.2017.0124

**Published:** 2018-08-06

**Authors:** Masumeh Ghazanfarpour, Mona Najaf Najafi, Nosrat Baharian Sharghi, Mahsa Sadat Mousavi, Masoudeh Babakhanian, Hassan Rakhshandeh

**Affiliations:** 1 Department of Midwifery, School of Nursing and Midwifery, Kerman University of Medical Sciences, Kerman, Iran; 2 Department of Community Medicine, Imam Reza Clinical Research Units, Mashhad University of Medicl Sciences School of Medicine, Mashhad, Iran; 3 Midwife, Omolbanin Hospital, Mashhad, Iran; 4 Department of Midwifery, Isfahan (Khorasgan) Branch, Islamic Azad University, Isfahan, Iran; 5 Social Determinant of Health Research Center, Semnan University of Medical Sciences, Semnan, Iran; 6 Pharmacological Research Center of Medicinal Plants, Mashhad University of Medical Sciences, Mashhad, Iran

**Keywords:** Foeniculum vulgare, menopausal symptoms, menopause-specific quality of life, post menopause, quality of life

## Abstract

**Objective:**

The present study aimed to evaluate the effects of oral fennel on menopausal symptoms.

**Material and Methods:**

This double-blind, randomized, placebo-controlled trial was conducted on 50 postmenopausal women in Mashhad (Iran). Patients were randomly divided into two groups of fennel (n=25) and placebo (n=25). Measurements were performed at baseline and after three months using the Menopause-Specific Quality of Life questionnaire.

**Results:**

Both placebo and treatment groups revealed significant improvements in the hot flush score (p<0.001 for fennel and p<0.01 for placebo), night sweats (p=0.007 for fennel and p<0.01 for placebo), sweating (p=0.002 for fennel and p<0.01 for placebo), symptoms of anxiety (p=0.05 for fennel and p=0.001 for placebo), feeling depressed (p<0.01 for fennel and p=0.006 for placebo), and impatience with other people (p<0.01 for fennel and p=0.003 for placebo). There were no significant differences in any menopausal symptoms between the fennel and placebo groups, except for coughing and sneezing when urinating (p=0.03).

**Conclusion:**

The failure to indicate a significant effect may have been caused by a high placebo response. It is suggested that future trials should include a placebo run-in phase or design a sequential, parallel study with larger sample sizes to mitigate the placebo effect.

## Introduction

All women experience the menopause as a normal phenomenon. Most menopausal women (nearly 80%) experience vasomotor and vaginal symptoms, urinary incontinence, joint pain, headaches, tachycardia, depression, dizziness, irregular heart rate, mental disorders, sexual dysfunction, and sleeplessness during their postmenopausal period. A few of these symptoms may last for several years (1,2,3). These complications impose a significant economic burden on society (4,5) and disrupt the normal life of people (1). A number of studies have revealed that hormone replacement therapy (HRT) prevents depression (6), sleep disorder (7), osteoporosis (8), and hot flashes (9); however, HRT is known to be associated with increased thrombosis and breast cancer (10). Therefore, postmenopausal women prefer to use nonhormonal compounds such as phytoestrogens, which offer a safer option (11,12,13). Phytoestrogens, as polyphenolic and nonsteroidal compounds in plants, can bind to human estrogen receptors; the effects of these compounds are less significant than those of endogenous steroidal estrogens. Based on *in vivo *and *in vitro* investigations, fennel, as a phytoestrogen, may treat several disorders including anxiety (14), depression, stress, sleep disorders (15), and vaginal atrophy (16), and various cognitive disorders such as Alzheimer’s disease and dementia (17). Accordingly, this study was designed to evaluate the role of fennel on attenuating the disorders associated with menopause.

## Material and Methods

Some menopausal symptoms, including quality of life and sleep, among postmenopausal women in Iran were studied following the oral consumption of fennel within the current randomized, double-blinded, placebo-controlled clinical trial. The Ethics Committee of Mashhad University of Medical Science approved the study protocol considering the principle of Declaration of Helsinki. This study continued for 17 months, from January 2015 to June 2016. All participants signed informed consent forms for their voluntary participation in the study, and they were allowed to leave the trial at any period. The inclusion criteria were healthy postmenopausal women with the age range of 45 to 65 years who had no vaginal bleeding in the previous year, a normal mammogram within the last year, and no history of taking systemic and tropical estrogen in the last six months. The subjects were selected from health centers in each area of Mashhad using the convenience sampling method. Therefore, Mashhad was divided into four geographic areas (north, south, west, and east) out of which 10 centers were randomly selected using cluster sampling. A list of menopausal women who were referred to the health centers was provided. All women on the list were contacted by telephone until 50 patients who met the eligibility criteria were selected. Participants were invited to the gynecology clinics of Game Hospital to complete a questionnaire.

### Sample size

The aim of this study was to investigate the impact of fennel on the quality of life among Iranian postmenopausal women. To this end, we found a study that assessed *Glycyrrhiza glabra* to determine the beneficial impact of this medicinal plant on quality of life in Iranian menopausal women. We chose this article because both fennel and *Glycyrrhiza glabra* are considered as phytoestrogens and contain flavonoids. Thus, the sample size was determined based on the difference between *Glycyrrhizin glabra* and placebo reported in the study of Asgari et al. (18) on vasomotor (*Glycyrrhiza glabra* mean ± SD= 1.23±1.07 and placebo mean ± SD=2.8±1.82), psychological (*Glycyrrhiza glabra* mean ± SD=3.8±1.9 and placebo mean ± SD=8.52±3.11), physical (red clover mean ± SD=7.6±3.91 and placebo mean ± SD=10.8±5.03) and sexual (*Glycyrrhiza glabra* mean ± SD= 7.29±6.15 and placebo mean ± SD=1.36±1.21) symptoms. The sample size was estimated using NCSS PASS software. With an alpha error of 0.2 and power of 80%, a sample size of n=10 for vasomotor, n=20 for physical, and n=7 for sexual was estimated.

### Measurements

The Menopause-Specific Quality of Life (MENQOL) questionnaire developed has three sections. Part I includes demographic information. Part II has researcher-made four items based on the literature to assess attitudes toward menopause (Are herbal medicines safer than chemical drugs? Are herbal medicines more effective than chemical drugs? Do you suggest using herbal therapy to address sexual issues for women? Do you think that herbal medicines can be harmful to your health?). Part III deals with 29 items within four subclasses, involving 3 vasomotor items, 7 psychosocial items, 16 physical items, and 3 sexual items. The scoring system of this questionnaire is based on a 6-point Likert scale ranging from 1 (no experience) to 5 (extremely bothersome), with higher scores indicating lower quality of life (19). The Persian version of this questionnaire has been validated by limited studies (20,21). The participants were asked to report the presence and severity of symptoms within the last month. If no (none), they continued to the subsequent item; if yes, they marked the severity of the symptom on a scale of 0-6. For participants who were illiterate, questions were read out by the interviewer and the responses were recorded.

### Randomization and blinding

Patients’ allocation sequencing was accomplished using a computerized random number generator. The study participants were randomly allocated to one of the two groups of fennel and placebo. To ensure that both patients and researchers were blinded to the treatment, capsules were identical in color (yellow), shape, and weight (100 mg capsules; as ensured by Barij Essence Company), and they all contained sunflower oil. Bottles contained high-density polyethylene and were labeled as “A” and “B.” All drugs were administered by assistant researchers who were not involved in the study. The identity of the bottles was not disclosed until the end of study.

### Intervention, compliance, and adverse event measures

Participants were instructed to consume capsules three times per day (morning, noon, and night) for a 3 month follow-up period. The soft 100 mg capsules contained 30% fennel (standardized to 21-27 mg anethole) supplemented with sunflower oil (http://www.barijessence.com). Compliance was checked by asking patients to bring unused capsules to each follow-up visit. Adverse events were investigated retrospectively based on the patient’s self-report.

### Statistical analysis

The obtained data were analyzed using SPSS 19 (SPSS Inc., Chicago, IL) using the Kolmogorov-Smirnov test to check data normality, and the chi-square test (for categorical data) and independent t-test (for continual data) to find the differences between the study groups, and the paired t-test to compare the results before and after the intervention. The significance level for all tests was considered to be p<0.05.

## Results

No adverse effects were reported in the study and there were no dropouts. The groups were comparable at baseline in terms of age, body weight, number of children, educational level, years of menopause, previous use of hormone therapy and vitamin supplement, use of herbal medicine, and cigarette smoking (Table 1). Both placebo and treatment groups revealed significant improvements in hot flush scores (p<0.01 for fennel and p<0.001 for placebo), night sweats (p=0.007 for fennel and p<0.01 for placebo), sweating (p=0.002 for fennel and p<0.001 for placebo), anxiety symptoms (p=0.05 for fennel and p<0.01 for placebo), feelings of depression (p<0.01 for fennel and p=0.006 for placebo), and impatience with other people (p<0.01 for fennel and p=0.003 for placebo). There was no significant difference between the two study groups. Sleep disorder scores were significantly reduced (2.3 points) on a 5-point scale (57%) in the fennel group, whereas this improvement was not significant in the placebo group (22%). The fennel group revealed a 43% decrease (improvement) in the severity of memory loss score, whereas this score increased (worsened) slightly (17%) in the placebo group. Surprisingly, placebo was found to have greater effect on the relief of depression (38% for fennel and 49% for placebo) (Table 2).

## Discussion

In this study, both fennel and placebo groups revealed a significant improvement in scores of hot flash, night sweats, sweating, anxiety, depression, and impatience with other people; however, the fennel group was not different from the placebo groups with respect to menopause symptoms. The high placebo response might have caused the negative results. A high placebo effect is often observed in psychiatric (22,23) and nonhormonal trials (24). Unexpectedly, the high placebo effect may be related to confounding factors such as patients’ expectations of treatment efficacy, past or current drug use, severity and duration of illness prior to the treatment response, and natural fluctuating patterns of the disease (23).

As shown in Table 1, when comparing the two groups, there was no significant difference in age, years of menopause, history of taking hormonal medications and vitamin supplements, and history of taking herbal medicine. The baseline attitudes toward herbal medicines showed no significant difference between the two groups. Moreover, no significant effect was observed in the subgroup high placebo (more than 50% vs less than 50%) in the placebo group; however, the limited attitude statements made us to interpret these findings cautiously.

It is assumed that fennel may improve memory and intelligence. Joshi and Parle (17) reported that methanolic extraction of fennel might have a memory-improving effect. This oral extraction at different concentrations of 50, 100, and 200 mg/kg was given to young mice for eight consecutive days. Fennel extract improved age-induced memory deficits. Moreover, fennel significantly increased step-down latency and acetyl cholinesterase inhibition. According to their conclusion, some cognitive disorders, including dementia and Alzheimer’s disease might be treated by the fennel extraction.

The memory-enhancing activity of fennel was assessed in an animal model using the conditioned avoidance response. Thus, the study rats separately experienced the training schedule. The rats were placed in the chamber. The pre-shock was a buzzer and then the main shock was exerted via the grid floor. To prevent the foot shock, the training program was a jump of the rats to the pole, i.e. shock-free zone. When exposed to the buzzer, the rats showed two responses: escape (if the rats jumped on the pole) or avoidance (if the rats jumped prior to the onset of the shock). The rats were classified into four groups of five. Groups II, III, and IV received methanolic fennel extract at concentrations of 50, 100, and 200 mg/kg, and group I was the controls. The fennel group had greater avoidance responses compared with the controls, whereas the control group showed higher amnesia. In addition, the amnesia was induced by scopolamine butyl bromide. It took 3-5 days for the fennel group and over 6 days for the control group to recover from the scopolamine-induced amnesia (25).

A study reported the sedative effect of 200 mg/kg aqueous extracts of fennel seeds in male albino rates. It also revealed a significant increase in some neurotransmitter content in all brain regions (26). Mesfin et al. (25) studied the anxiolytic activity of *Foeniculum vulgare* essential oil on a murine model. The administration of fennel decreased anxiety and depression by 55% and 45% compared with baseline, respectively. The data from *in vitro* and *in vivo* studies suggest anti-anxiety and anti-depression effects of the fennel extract.

Shirazi et al. (15) demonstrated the impacts of fennel combined with officinalis (Melissa) on improving the Pittsburgh Sleep Quality Index among menopausal women with sleep disorders. A significant improvement was observed in the sleep disorders in the Melissa group compared with the citalopram and placebo groups. Consistent with the study of Shirazi et al. (15), this study mitigated sleep disorder by 57%. This improvement in sleep disorder could be related to the sedative effect of fennel.

In a trial by Yaralizadeh et al. (16), 60 postmenopausal women were randomly assigned to fennel 5% vaginal cream (5 g/day) and placebo vaginal cream for eight weeks. All symptoms such as itching (p=0.017), dryness (p<0.001), and pallor (p<0.001), with the exception of burning (p=0.14), improved significantly compared with placebo. In accordance with the study of Yaralizadeh et al. (16), our data suggested a 50% decline in vaginal dryness.

In an Iranian trial, 38 women were randomly assigned to 3 groups of 1% and 2% fennel cream, and placebo. Hair thickness plummeted in a dose-dependent manner from 7.8% with 1% cream to 18.3% with the 2% cream (27). Akha et al. (28) in Iran randomized 42 women with mild-to-moderate hirsutism into 3% fennel gel and placebo groups. After 24 weeks, the fennel group showed a significant decrease in hair thickness (from 97.9±31.5 to 75.6±26); there was no change in the placebo group.

Surprisingly, women in the placebo group of the present study reported a 56% decline compared with the fennel group (26%) in “body weight gain” symptoms, although the difference between the groups was nonsignificant. This is in contrast to other human and animal studies. Fennel, both as tea and aromatherapy, could suppress appetite in women with excess weight (29,30). Hur et al. (31) reported similar results in animal models. Their study indicated no significant reduction in body weight of rats. However, the fennel group had a lower rate of food efficacy when compared with the other groups. Nevertheless, the results should be interpreted cautiously because the fennel effect on body weight was assessed using subjective symptoms; however, the unpublished findings of a study on the effect of fennel on the lipid profile did not alter the body weight at the end of 3-months follows-up.

The main limitation of this study was the high placebo response observed in all symptoms. The results of a post analysis power calculation indicated that the present sample size was insufficient for all parameters of the MENQOL questionnaire.

The failure to indicate a significant effect may be due to the high placebo response. It is suggested that future trials include a placebo run-in phase or design a sequential, parallel study or with a longer follow-up period with larger sample sizes to mitigate the placebo effect.

## Figures and Tables

**Table 1:**
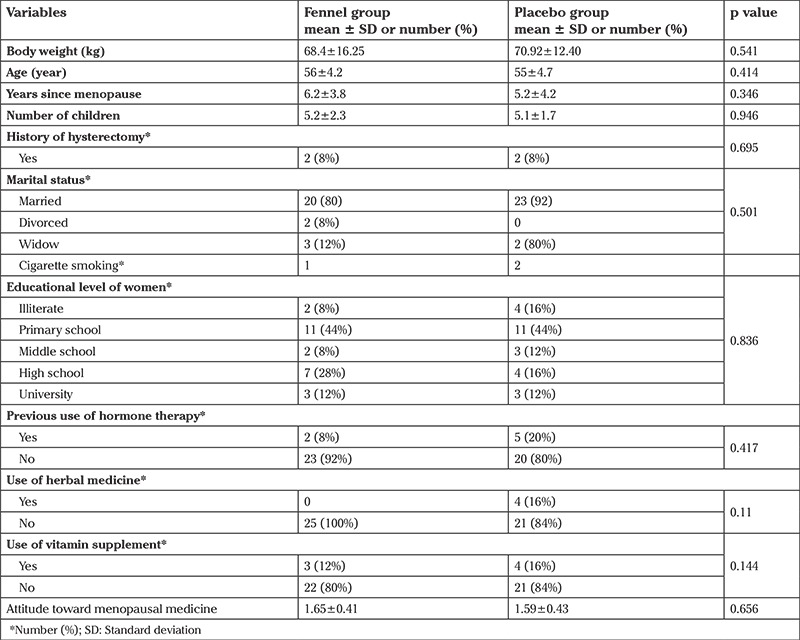
The demographics and baseline characteristics of subjects in the two groups

**Table 2:**
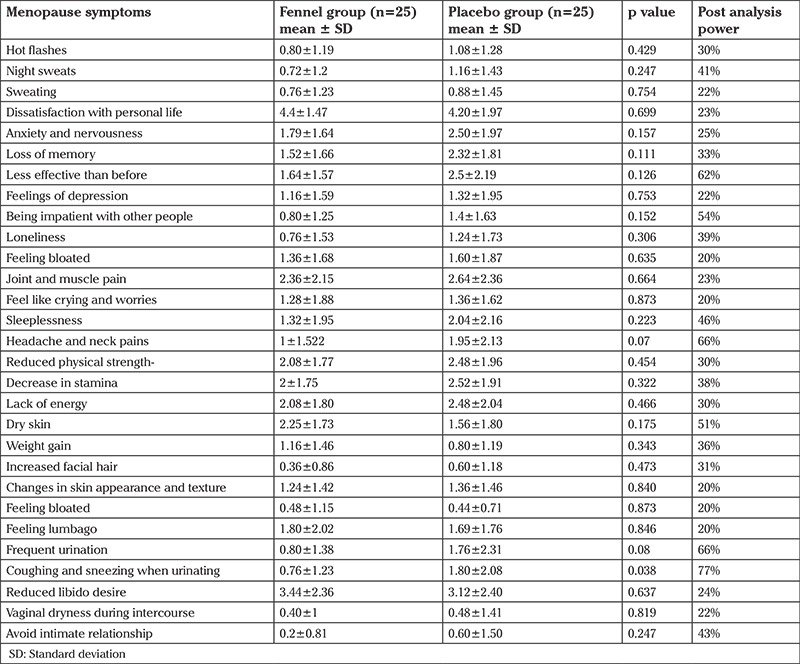
Comparison of menopausal symptoms between two groups
